# The complete chloroplast genome sequence of *Swertia diluta* (Gentianaceae)

**DOI:** 10.1080/23802359.2020.1788447

**Published:** 2020-07-11

**Authors:** Zhen Yang, Jinyan Yan, Guoyu Zhu

**Affiliations:** a Key Laboratory of Bio-Resource and Eco-Environment of Ministry of Education, College of Life Sciences, Sichuan University, Chengdu, Sichuan, P. R. China; b Aba Vocational College, Mao County, Sichuan, P. R. China; c State Key Laboratory of Hydraulics and Mountain River Engineering, College of Water Resource and Hydropower, Sichuan University, Chengdu, Sichuan, P. R. China

**Keywords:** *Swertia diluta*, Gentianaceae, chloroplast genome, phylogenetic tree

## Abstract

*Swertia diluta*, a traditional Chinese medicine, is widely used to treat jaundice hepatitis, dysentery, dyspepsia, etc. The plastome is 153,691 bp in length, with one large single copy region of 83,860 bp, one small single copy region of 18,301 bp, and two inverted repeat (IR) regions of 25,765 bp. It contains 134 genes, including 84 protein-coding genes, 8 ribosomal RNA, and 37 transfer RNA. Phylogenetic tree shows that *S. diluta* is a sister species to *S. mussotii*. The complete chloroplast genome could provide genetic information of this species would contribute to the formulation of protection strategy.

*Swertia diluta* (Turcz.) Benth. et Hook. f. belongs to *Swertia* (Gentianaceae), is a traditional Chinese medicine (Ho and Liu 2001), This medicinal plant is widely used to treatment jaundice hepatitis, dysentery, dyspepsia and other diseases. However, due to anthropogenic over exploitation and decreasing distributions, this species needs urgent conservation. Knowledge of the genetic information of this species would contribute to the formulation of protection strategy (Xiao et al. 2010). As one of important target for genetic transformation, the full chloroplast genome could supply more genetic information. So far, the chloroplast genome of *S. diluta* has not been reported. In this study, we reported the complete chloroplast genome sequence of *S. diluta* based on the next-generation sequencing, and the annotated genomic sequence was submitted to GenBank.

Fresh leaves of *S. diluta* were collected from Nanhuashan mountain (Zhongwei, Ningxia, China; coordinates: 105°38′E, 36°21′N) and dried with silica gel. The voucher specimen was stored in Sichuan University Herbarium with the accession number of QTPLJQ13383005. Total DNA was isolated using a modified CTAB method (Doyle and Doyle 1987) and sequenced by the BGISEQ-500 sequencing platform (BIG, Shenzhen, China). A total 10 million high quality pair-end reads were used to assemble the complete chloroplast genome by NOVOPlasty (Dierckxsens et al. 2017). The complete chloroplasts genome sequence of *S. leducii* was used as a reference. Plann v1.1 (Huang and Cronk 2015) and Geneious v11.0.3 (Kearse et al. 2012) were used to annotate the chloroplasts genome and correct the annotation.

The total plastome length of *S. diluta* (MT588299) is 153,691 bp, exhibits a typical quadripartite structural organization, consisting of a large single copy (LSC) region of 83,860 bp, two inverted repeat (IR) regions of 25,765 bp, and a small single copy (SSC) region of 18,301 bp. The cp genome contains 134 complete genes, including 84 protein-coding genes (84 PCGs), eight ribosomal RNA genes (4 rRNAs), and 37 tRNA genes (37 tRNAs). Most genes occur in a single copy, while 14 genes occur in double, including seven tRNAs (*trnA-UGC*, *trnI-CAU*, *trnI-GAU*, *trnL-CAA*, *trnN-GUU*, *trnR-ACG*, and *trnV-GAC*), and seven PCGs (*rps7*, *rps19*, *rpl2*, *rpl23*, *ndhB*, *ycf15*, *ycf2*), while one partial *rps19* gene was identified as a pseudogene. The overall AT content of cp DNA is 61.9%, the corresponding values of the LSC, SSC, and IR regions are 63.8%, 68.1%, and 56.5%.

To confirm the phylogenetic location of *S. diluta* within the family of Gentianaceae, a total of 10 complete cp genomes of Gentianaceae were obtained from GenBank, and *Catharanthus roseus* was used as out-group. All the sequences were aligned using MAFFT v.7.313 (Katoh and Standley 2013) and maximum likelihood phylogenetic analyses were conducted using RAxML v.8.2.11 (Stamatakis 2014). The phylogenetic tree shows that all species were identified two clades. *G. ornata*, *G. caelestis*, *G. straminea*, *G. crassicaulis* and *G. tibetica* together one clustered. Remian species together another clustered, While *S. diluta* is a sister species to *S. mussotii* in this clade (Figure 1).

**Figure 1.**
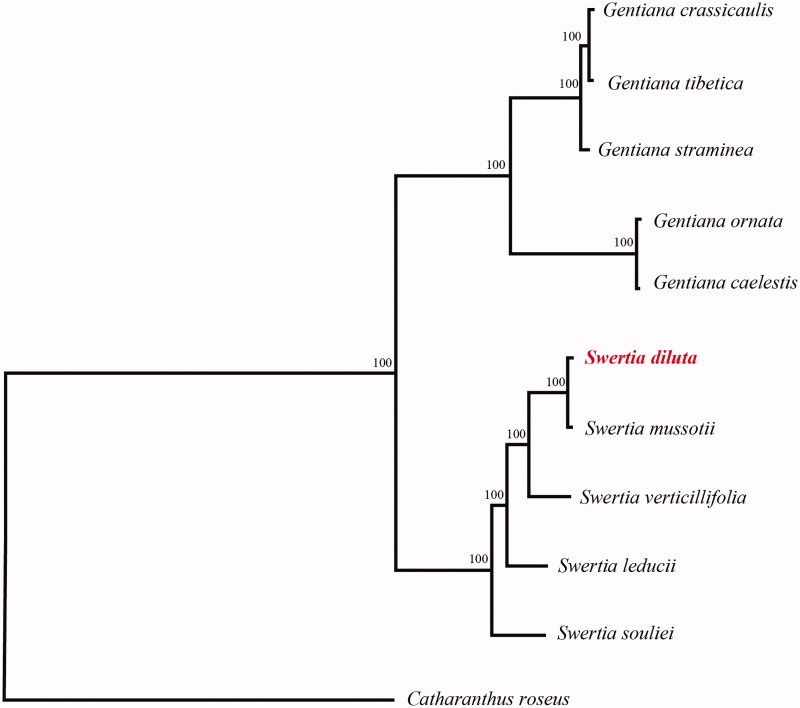
A plastome phylogenetic tree of Gentianaceae species. GenBank accession numbers: *Swertia diluta* (MT588299); *Swertia souliei* (MT185926); *Swertia leducii* (NC_045301); *Swertia verticillifolia* (MF795137); *Swertia mussotii* (NC_031155); *Gentiana ornata* (NC_037983); *Gentiana caelestis* (MG192304); *Gentiana straminea* (KJ657732); *Gentiana crassicaulis* (NC_027442); *Gentiana tibetica* (NC_030319); *Catharanthus roseus* (NC_021423).

## Data Availability

The data that support the findings of this study are openly available in GenBank of NCBI at https://www.ncbi.nlm.nih.gov, reference number MT588299.
